# Efficacy and Safety of Psychedelics in Treating Anxiety Disorders

**DOI:** 10.31486/toj.23.0076

**Published:** 2023

**Authors:** Leah Feulner, Thanpicha Sermchaiwong, Nathan Rodland, David Galarneau

**Affiliations:** ^1^ The University of Queensland Medical School, Ochsner Clinical School, New Orleans, LA; ^2^ Department of Psychiatry, Ochsner Clinic Foundation, New Orleans, LA

**Keywords:** *Agents–psychedelic*, *anxiety*, *ayahuasca*, *hallucinogens*, *ketamine*, *LSD*, *MDMA*, *psilocybin*

## Abstract

**Background:** Anxiety disorders are commonly diagnosed and cause substantial functional impairment. A mixture of pharmacologic and psychosocial treatments currently exists, but these treatments are not always tolerable and effective. For patients with anxiety resistant to standard therapy, psychedelics may be a promising alternative. This review assesses the therapeutic benefits and safety of psychedelics in treating anxiety disorders.

**Methods:** We searched PubMed, Embase, PsycInfo, and CINAHL for clinical trials investigating psychedelics in patients with clinician-diagnosed generalized anxiety disorder, social anxiety disorder, specific phobia, separation anxiety disorder, selective mutism, panic disorder, agoraphobia, and anxiety attributable to another medical condition. We analyzed data from 9 independent psychedelic-assisted trials testing ayahuasca (1 study), ketamine (4 studies), lysergic acid diethylamide (LSD) (2 studies), 3,4-methylenedioxymethamphetamine (MDMA) (1 study), and psilocybin (1 study). Efficacy was assessed by measuring the change in outcome measures and the quality of life from baseline.

**Results:** The reviewed studies demonstrated encouraging efficacy in reducing anxiety symptoms, increasing self-perception, and increasing social function in patients with generalized anxiety disorder, social anxiety disorder, or anxiety attributable to another medical condition while establishing feasibility and evidence of safety. For many patients, the therapeutic effects of the psychedelic treatment lasted weeks, and no severe adverse events were reported.

**Conclusion:** Based on the evidence of symptom reduction and safety, the current literature (2011 to 2021) shows that psychedelics could be considered for treating clinician-diagnosed anxiety disorders. Psychedelics may provide an alternative therapeutic option for patients resistant to current standard treatments.

## INTRODUCTION

Anxiety disorders are among the most prevalent psychiatric disorders worldwide, causing significant impacts on physical and emotional health and substantial functional impairment.^1,2^ Approximately 33.7% of individuals in the United States between the ages of 13 and 64 have been diagnosed with an anxiety disorder at some point in their lives.^3^ In the *Diagnostic and Statistical Manual of Mental Disorders* (Fifth Edition, Text Revision) (DSM-5-TR), anxiety disorders include generalized anxiety disorder, social anxiety disorder, separation anxiety disorder, selective mutism, specific phobia, panic disorder, agoraphobia, substance-/medication-induced anxiety disorder, anxiety disorder attributable to another medical condition, other specified anxiety disorder, and unspecified anxiety disorder.^4^ Anxiety disorders are characterized by intense and excessive fear and worry, as well as maladaptive behavioral changes such as avoidance behavior.^5,6^ Patients with an anxiety disorder can present with a wide range of cognitive and somatic symptoms depending on the type of disorder, such as persistent fear about specific situations, nausea, and palpitations.^6^ Symptoms can range in severity from mild to severe and can cause significant functional impairment.

Anxiety disorders often have a substantial negative impact on an individual's quality of life.^7^ On a societal level, anxiety disorders have an associated economic burden, as some patients have difficulty working and effectively performing in their jobs and may require mental health treatment and services.^8^ A 1996 study conducted on the economic cost of anxiety disorders using the human capital approach estimated that the combined direct and indirect costs associated with anxiety disorders were $46.6 billion US dollars in 1990.^9^

The natural course of anxiety is akin to other chronic medical illnesses. Despite numerous available pharmacologic and nonpharmacologic treatments, anxiety disorders are often chronic, with fluctuating symptom severity.^10,11^ Complete remission is uncommon.^10,11^ Furthermore, anxiety disorders are complicated by high comorbidity with other psychiatric disorders, such as major depressive disorder,^1,12^ and are often associated with a higher risk of medical comorbidities (including cardiovascular events) and poorer health outcomes compared to the general population.^13^

Although pharmacologic agents and psychotherapies are frequently used to treat anxiety disorders, these treatments are not always tolerated or effective.^14-16^ While cognitive behavioral therapy is considered an effective therapy for anxiety disorders, some patients do not improve with treatment, some are unwilling to participate, others do not have access to a cognitive behavioral therapy–based therapist, and some patients are unable to afford the therapy.^14,17,18^

Selective serotonin reuptake inhibitors (SSRIs) and serotonin and norepinephrine reuptake inhibitors (SNRIs) are first-line pharmacologic agents for the treatment of anxiety disorders,^19^ but some patients do not respond adequately to treatment with these agents and may develop a treatment-resistant anxiety disorder.^14,20,21^ SSRIs can take 4 to 6 weeks to achieve symptom amelioration and often have significant adverse effects, including withdrawal symptoms, nausea, increased anxiety with initial use, sexual dysfunction, and insomnia.^15^ These adverse reactions and delayed effects often lead to nonadherence.^22^

Psychedelics are substances that alter cognition, mood, and sensory perception.^23,24^ Some plant-based psychedelics, such as ayahuasca, peyote, and psilocybin, are entheogens, psychoactive substances specifically used in religious and spiritual contexts.^25-27^ Entheogens have a long history; plant-based psychedelics have been used worldwide for centuries for ritual and healing purposes.^23,26^ In modern Western civilization, however, the therapeutic use of psychedelics began with the discovery of lysergic acid diethylamide (LSD) by chemist Albert Hofmann at Sandoz in 1943.^28^ Research into the therapeutic use of psychedelics to treat psychiatric disorders bloomed in the United States in the 1950s and 1960s, with numerous studies published about their potential to treat a wide range of diagnoses such as alcoholism, addiction, depression, and anxiety.^29-31^ Not only were psychedelics investigated for their therapeutic potential but also as a tool for understanding the pathogenesis of psychiatric illnesses.^30^

The therapeutic use of psychedelics was principally investigated using 2 models: the psycholytic model and the psychedelic model.^30,32^ The psycholytic model used several low doses of psychedelics in combination with psychoanalytic therapy; the psychedelics were thought to enhance the efficacy of psychotherapy by deepening the experience and accessing the subconscious.^30,32,33^ The psychedelic model used higher doses of psychedelics to induce a mystical experience; this therapeutic use was commonly studied in patients with alcoholism.^30,32,33^ However, research into psychedelic therapies slowed with the Controlled Substances Act of the Comprehensive Drug Abuse Prevention and Control Act of 1970.^32^ As a result, LSD and other psychedelics became classified as Schedule 1 drugs, the classification given to drugs that the US government considers to lack an acceptable use in medicine and to have a significant potential for harm and abuse.^34^

Further exploration of the therapeutic and recreational potential of psychedelics continued underground despite the new regulations.^35-37^ Renewed interest in the continuation of legitimate psychedelic research began with the development of modern neuroimaging techniques and the enhanced understanding of the neurobiology of psychiatric disorders.^23,38^ Since then, studies have been conducted into the therapeutic efficacy and safety profile of specific psychedelics for various conditions such as anxiety, posttraumatic stress disorder, depression, smoking, and treatment-resistant obsessive-compulsive disorder.^23^ Many of these studies supported the efficacy of psychedelic substances in improving symptoms and demonstrated that the substances were generally well tolerated.^23,32^

Substances included under the umbrella term *psychedelics* have a wide range of mechanisms of action. Broadly speaking, *classic psychedelics* refer to substances that produce their psychotropic effects primarily by acting as agonists or partial agonists of 5-hydroxytryptamine (serotonin) type 2A (5-HT2A) receptors.^39-41^ However, psychedelics have also been shown to interact with other receptors such as 5-HT1A, 5-HT2B, and 5-HT2C.^42^ LSD, psilocybin, ayahuasca (a plant-based decoction that contains beta-carboline alkaloids and dimethyltryptamine), mescaline, peyote (a cactus that contains mescaline), and 2,5-dimethoxy-4-bromophenethylamine (2C-B, a synthetic drug similar in structure to mescaline) are all classic psychedelics.^43,44^ These psychedelics can produce changes in mood, thinking patterns, and sensory perception and can cause visual and auditory hallucinations, dissociation, and mystical experiences.^41^ The exact mechanism of how these substances produce therapeutic effects for psychiatric disorders remains unclear.^29^ Psychedelics may directly affect neuronal connections and brain activity or exert effects as an adjunct to traditional psychotherapy.^29^

Recent (2023) research performed on mice demonstrated that psychedelics may produce a metaplastic change in the brain.^45^ Metaplasticity is the extent to which synaptic plasticity can be initiated.^46^ The induction of synaptic plasticity suggests the ability to reopen the critical period of learning, allowing increased psychological flexibility and cognitive reappraisal.^45^ These properties have been linked to successful treatment of anxiety.^46^ Psychologically, psychedelics have been demonstrated to work through ego dissolution, the separation of boundaries between the world and oneself.^47^ After the onset of ego dissolution, psychedelic experiences are thought to create personal insight through introspection by removing mental hurdles and relaxing the psychological or ego resistance.^48,49^ Allowing relaxation of the maladaptive beliefs helps dissolve rigid and ingrained patterns of thinking.^49^

Nonclassic psychedelics are other substances that can alter cognition and sensory perception and produce a psychedelic-like experience; this category includes 3,4-methylenedioxymethamphetamine (MDMA), ketamine, and salvia.^43^ These substances have mechanisms of action distinct from those of classic psychedelics. For instance, MDMA, also known as the street drug ecstasy, works primarily by releasing serotonin, norepinephrine, and dopamine.^50^ Studies have shown that MDMA can produce empathogenic feelings such as empathy, kindness, and connection to others and increase social approach behavior.^51^ MDMA-assisted psychotherapy has been used to treat certain psychiatric disorders, particularly posttraumatic stress disorder.^52^ Ketamine, a dissociative anesthetic, can similarly alter levels of consciousness and is hypothesized to assert an effect as an antidepressant via its N-methyl-D-aspartate receptor antagonist action.^53^ In a small study, Berman et al showed that ketamine had rapid antidepressant effects in patients with major depressive episodes, and significant improvement in symptoms occurred within hours, making ketamine a substance of particular interest for treating depression.^54^ Salvia (*Salvia divinorum*) is an herb native to Mexico that produces its hallucinogenic effects via the ingredient salvinorin A that activates kappa opioid receptors.^55^ Like ketamine, salvinorin A has been identified as a potential candidate for major depressive disorder treatment research.^56^

Psychedelic compounds have been recognized as beneficial in treating certain psychiatric disorders. For example, in January 2023, Oregon became the first US state to legalize the therapeutic use of psilocybin mushrooms.^57^ Australia's medical regulator announced that as of July 1, 2023, authorized psychiatrists may prescribe MDMA for the treatment of posttraumatic stress disorder and psilocybin for treatment-resistant depression.^58^ Entheogens, particularly psilocybin, have been decriminalized in some cities and states in the United States or have active governmental bills regarding decriminalization.^59^ Despite the therapeutic potential of psychedelic-assisted therapy for a variety of psychiatric conditions, many challenges complicate the use of psychedelic-assisted therapies in a clinical setting, including the cost of treatments and ethical and legal restrictions.^35^

Although studies have investigated the efficacy of psychedelics in treating patients with psychiatric disorders such as posttraumatic stress disorder, major depressive disorder, and obsessive-compulsive disorder with anxiety either coexisting with the diagnosis or as a comorbidity, less research has examined the effect of psychedelics on patients with anxiety disorders as the primary diagnosis. This review focuses on the therapeutic efficacy and safety profile of psychedelic-assisted therapy in patients with anxiety disorders.

## METHODS

The literature review was conducted and reported according to the Preferred Reporting Items for Systematic Reviews and Meta-Analyses (PRISMA) guidelines.^60^

### Studies, Participants, and Interventions

For this original literature review, we searched for clinical trials and case reports investigating the effects of ayahuasca, peyote, ketamine, LSD, MDMA, psilocybin, mescaline, salvia, and 2C-B on anxiety symptoms that were published in peer-reviewed journals. We limited publications to papers in the English language, and we required access to the entirety of the publication. Posters, reviews, abstracts, and animal studies were excluded. Patients included in the studies must have had an established diagnosis of one of the DSM-IV-TR, DSM-5, or DSM-5-TR anxiety disorders.

### Outcome Measures

The efficacy of the psychedelics on anxiety symptoms was measured using different scales and self-reported questionnaires. Symptom improvement was measured by comparing baseline scores to scores at various follow-up periods. The safety and tolerability of the psychedelics were investigated by noting the incidence and types of adverse effects.

### Search Methods and Extraction

Two authors (LF and TS) conducted the literature search in August 2022 in the PubMed, Embase, PsycInfo, and CINAHL online databases using the search words and terms LSD, psilocybin, ayahuasca, MDMA, mescaline, peyote, ketamine, salvia, 2C-B, “separation anxiety,” “generalized anxiety disorder,” “social anxiety,” “selective mutism,” “panic disorder,” “agoraphobia,” and “anxiety.” No time frame was defined for excluding articles. All publications were downloaded into EndNote (Clarivate Plc), and duplicates were removed. All publications were uploaded into Rayyan (Rayyan Systems, Inc) to be screened by title and abstract by 3 authors (LF, TS, and NR). The authors (LF, TS, and NR) reviewed the remaining full-text articles based on the eligibility criteria. The reference lists of notable reviews were also manually searched for relevant articles.

Authors, publication date, patient diagnosis, sample size, study design, research question, psychedelic intervention, follow-up, response, and type of outcome measure were extracted using Qualtrics (Qualtrics).

### Bias Assessment

The Cochrane risk-of-bias tool^61^ was used to investigate each manuscript for bias. This tool covers 6 bias domains: selection bias, performance bias, detection bias, attrition bias, reporting bias, and other bias. Bias in each of these individual domains was assessed and categorized into 3 categories:

1. Low risk: plan to reduce bias is distinctly defined and good.
2. Unclear risk: plan to reduce bias is not mentioned, or bias effects are unknown.
3. High risk: plan to reduce bias is absent or inadequate.

The assessment of whether the risk in each domain was high or low depended on whether bias of a sufficient magnitude was found to have influenced the results or the conclusions of the trial. If the trial had no mention of a particular domain or an insufficient description of the process, the domain risk was categorized as unclear. To promote transparency regarding each risk assessment, detailed information relevant to each of the bias domains from the trials included in our review is presented in the Appendix.

We chose to use the Cochrane risk-of-bias tool to provide a standardized way for readers to quickly appraise the reliability of each trial. Author NR performed the bias assessment. NR consulted with authors LF and TS if the bias determination was not clear-cut, and all 3 authors conferred until a decision was made.

## RESULTS

### Search Results

The literature search yielded 216 database records (Figure). We eliminated 27 duplicates and screened 189 titles and abstracts. Of these records, 169 records were excluded, and 20 full-text articles were sought for retrieval to determine eligibility. One full-text article could not be retrieved, and 11 articles were further excluded. One additional article was identified via manual searching of reference lists in cited articles. Nine studies were included in this review.

**Figure.**
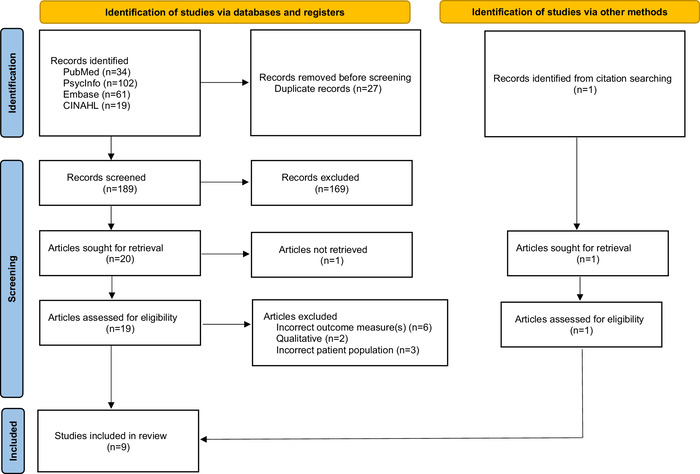
Preferred Reporting Items for Systematic Reviews and Meta-Analyses (PRISMA) Flow Diagram.

Studies were classified according to drug and anxiety disorder (generalized anxiety disorder, social anxiety disorder, anxiety associated with life-threatening disease). Study inclusion was based on whether the study included a diagnosed anxiety disorder without coexisting major depressive disorder. Three studies^62-64^ controlled for depression. The other 6 studies^65-70^ did not control for depression but were included because major depressive disorder was not costudied or mentioned as a current coexisting disease. Of the included studies, 1 used ayahuasca^70^ (for social anxiety disorder), 4 used ketamine^62-64,68^ (for treatment-resistant generalized anxiety disorder or social anxiety disorder), 2 used LSD^66,67^ (for anxiety associated with life-threatening illness), 1 used MDMA^65^ (for anxiety associated with life-threatening illness), and 1 used psilocybin^69^ (for anxiety associated with life-threatening cancer and generalized anxiety disorder). Details of the included studies are summarized in Table 1. We did not find any studies examining the effects of peyote, 2C-B, mescaline, or salvia on anxiety disorders.

**Table 1.** Summary of Clinical Trials Assessing the Anxiolytic Effects of Ayahuasca, Ketamine, LSD, MDMA, and Psilocybin

Study	N/Diagnosis	Study Design	Drug and Dose	Principal Findings
Dos Santos et al, 2021^70^	17/SAD	Double-blind, placebo-controlled, randomized group trial	Ayahuasca 2 mL/kg	Significant reduction in the SSDPS scale with no effect of time; increased self-perception and speech performance at days 7, 14, and 21; anxiety scores via VAMS and BAI were not significantly decreased at days 7, 14, and 21
Glue et al, 2017^62^	12/Treatment-resistant GAD or SAD	Uncontrolled, open-label trial	Subcutaneous injection of ketamine 0.25 mg/kg, 0.5 mg/kg, and 1 mg/kg	50% reduction in HAM-A and FQ scores within 1 hour after 0.5 or 1 mg/kg dose that persisted for up to 7 days
Glue et al, 2020^64^	12/Treatment-resistant GAD or SAD	Double-blind, controlled trial	Subcutaneous injection of ketamine 0.25 mg/kg, 0.5 mg/kg, and 1 mg/kg	Majority of patients reported >50% decrease in HAM-A and FQ scores after 0.5 or 1 mg/kg dose
Glue et al, 2018^63^	20/Treatment-resistant GAD or SAD	Uncontrolled, open-label trial	Subcutaneous injection of ketamine 1 mg/kg	50% reduction of HAM-A and FQ scores within 1 hour of administration
Taylor et al, 2018^68^	18/SAD	Double-blind, randomized, controlled crossover trial	IV ketamine 0.5 mg/kg	Significant reduction in LSAS scores compared to placebo at 2- and 10-day follow-up; no significant change from placebo on the VAS for anxiety symptoms
Gasser et al, 2014^67^	12/Anxiety associated with life-threatening illness	Double-blind, randomized, placebo-controlled trial	LSD 200 μg	Significant reduction in STAI-S scores at the 2-month follow-up and sustained after 12 months; STAI-T scores were not significantly decreased
Gasser et al, 2015^66^	10/Anxiety associated with life-threatening illness	Open-label, nonplacebo trial	LSD 200 μg	Significant reduction in STAI-S and STAI-T scores at 12-month follow-up, 77.8% reported sustained reduction in anxiety
Wolfson et al, 2020^65^	18/Anxiety associated with life-threatening illness	Double-blind, randomized, placebo-controlled trial	MDMA 125 mg	Reduction in STAI-T scores by –23.5 at 1 month after second psychotherapy session; statistically significant reductions of STAI-T and STAI-S at 6- and 12-month follow-up
Grob et al, 2011^69^	12/Anxiety associated with advanced-stage cancer, GAD	Double-blind, randomized, controlled trial	Psilocybin 0.2 mg/kg	Significant reduction in STAI-T scores for the entire 6-month follow-up

BAI, Beck Anxiety Inventory; FQ, Fear Questionnaire; GAD, generalized anxiety disorder; HAM-A, Hamilton Anxiety Rating Scale; IV, intravenous; LSAS, Liebowitz Social Anxiety Scale; LSD, lysergic acid diethylamide; MDMA, 3,4-methylenedioxymethamphetamine; SAD, social anxiety disorder; SSDPS, Self-Statements During Public Speaking; STAI-S, State-Trait Anxiety Inventory-State; STAI-T, State-Trait Anxiety Inventory-Trait; VAMS, visual analog mood scale; VAS, visual analog scale.

### Ayahuasca for Social Anxiety Disorder

A 2021 proof-of-concept, double-blind, placebo-controlled, randomized parallel-group trial examined the anxiolytic effects and change in self-perception after 1 dose (2 mL/kg) of ayahuasca compared to placebo in 17 patients (2 males, 15 females) with social anxiety disorder.^70^ Although the ayahuasca group had decreased visual analog mood scale anxiety scores, ayahuasca did not produce significantly decreased visual analog mood scale anxiety scores at the 3 follow-up appointments (days 7, 14, and 21) compared to placebo. No significant difference in anxiety as assessed by the Beck Anxiety Inventory was seen between groups. On the Self-Statements During Public Speaking scale, patients receiving ayahuasca had significant increases in self-perception and speech performance at all 3 follow-ups.^70^

### Ketamine for Treatment-Resistant Generalized Anxiety Disorder or Social Anxiety Disorder

Glue et al conducted an uncontrolled, open-label trial examining the effect of ascending single doses (0.25, 0.5, and 1 mg/kg) of ketamine on anxiety ratings in 12 patients (8 male, 4 female) with treatment-resistant generalized anxiety disorder or social anxiety disorder.^62^ The study was published in 2017. All patients had a Hamilton Anxiety Rating Scale (HAM-A) score >20 or a Liebowitz Social Anxiety Scale (LSAS) score >60. To avoid confounding results of comorbid depression, subjects with Montgomery-Asberg Depression Rating Scale (MADRS) scores >20 were excluded. Fear Questionnaire (FQ) scores showed a significant dose × time interaction. HAM-A scores showed an initial decrease and duration of reduced scores but not a statistically significant difference from baseline at the 7-day follow-up. Ten of the 12 patients reported a >50% reduction in HAM-A and FQ scores after the 0.5 or 1 mg/kg dose.

A double-blind, psychoactive-controlled ascending dose study by Glue et al (published in 2020) examined the effect of ascending single doses (0.25, 0.5, and 1 mg/kg) of ketamine on anxiety ratings compared to placebo in 12 patients (8 male, 4 female) with treatment-resistant generalized anxiety disorder or social anxiety disorder.^64^ All patients had a HAM-A score >20 and/or an LSAS score >60. To avoid confounding results of comorbid depression, subjects with MADRS scores >20 were excluded. The mean HAM-A at screening was 28.1, and the mean LSAS at screening was 91.3. Midazolam (0.01 mg/kg), a potent anxiolytic, was used as the placebo. The placebo effect mirrored the effects of the 0.25 mg/kg dose, but analysis of variance (ANOVA) showed a significant dose × time effect on FQ scores. The effects of midazolam overlapped the change in FQ scores for the 0.5 mg/kg ketamine dose, but ANOVA showed a significant effect of HAM-A vs dose and HAM-A vs time. Eight of the 12 patients reported a >50% decrease in HAM-A and FQ scores after the 0.5 or 1 mg/kg doses.

In 2018, Glue et al published an uncontrolled open-label study that examined the change in anxiety ratings, tolerability, and anxiety remission after 3 months of 2 weekly ketamine injections (1 mg/kg) in 20 patients (10 male, 10 female) with treatment-resistant generalized anxiety disorder or social anxiety disorder.^63^ The participants in this study had already been deemed as responders in the preliminary ascending dose-finding trials (published in 2017^62^ and 2020^64^). The mean HAM-A score of these patients at baseline was 12.6, a reduction from the previous mean of 25 because of their participation in one of the original ascending dose trials. An hour after the ketamine injection, FQ and HAM-A ratings decreased by 50%. FQ scores reached a plateau by 7.5 weeks, and HAM-A scores plateaued by 3.5 weeks. Most participants reported an increased ability to concentrate and perform everyday tasks.^63^

### Ketamine for Social Anxiety Disorder

A 2018 double-blind, randomized, placebo-controlled crossover trial examined the effects of intravenous (IV) ketamine (0.5 mg/kg over 40 minutes) vs placebo (normal saline) on social phobia symptoms in 18 adults (11 male, 7 female) with social anxiety disorder.^68^ Each patient either received ketamine or placebo on day 0 and then received the other infusion on day 28. Patients receiving ketamine demonstrated a significant decrease in LSAS scores compared to placebo at the 2- and 10-day follow-ups but did not show a significant change from placebo for the anxiety symptoms on the visual analog scale.^68^

### LSD for Anxiety Associated With Life-Threatening Illness

A 2014 double-blind, randomized, placebo-controlled pilot study examined the safety and efficacy of LSD-assisted psychotherapy in 12 patients (8 male, 4 female) on anxiety associated with life-threatening illness.^67^ All 12 patients reported a baseline State Trait Anxiety Inventory-State (STAI-S) or State Trait Anxiety Inventory-Trait (STAI-T) score >40. STAI scores were compared before and after 2 experimental psychotherapy sessions with 200 μg of LSD or placebo (20 μg of LSD) 2 to 3 weeks apart. All participants were followed for 12 months after the study. STAI-S scores were significantly reduced at the 2-month follow-up and were sustained after 12 months, but the STAI-T scores were not significantly decreased. Nonanxiety measures also had significant outcomes, including the 30-item European Cancer Quality of Life Questionnaire, the Hospital Anxiety and Depression Scale, and the Symptom Checklist-90-Revised.^67^

At the conclusion of the study, participants who received placebo were offered open-label treatment with 200 μg of LSD. The long-term follow-up, open-label, nonplacebo clinical trial (2015) included 10 (6 males, 4 females) of the original 12 participants^66^; 3 of the participants received placebo in the original clinical trial.^67^ The new study looked at the long-term effect on STAI anxiety scores after 3 months of treatment with 6 to 8 psychotherapy sessions and 2 LSD experiences (200 μg) at a 4- to 6-week interval. STAI-S and STAI-T scores showed significant reductions at the 12-month follow-up. Semistructured interviews conducted with the participants and evaluated via qualitative content analysis concluded that 77.8% reported sustained reductions in anxiety, 77.9% reported less fear of death, and 66.7% reported improved quality of life.^66^

### MDMA for Anxiety Associated With Life-Threatening Illness

A 2020 double-blind, randomized, placebo-controlled trial examined the change in anxiety ratings before and after administration of MDMA (125 mg) with two 8-hour psychotherapy sessions in 18 patients (4 male, 14 female) with anxiety associated with life-threatening illness.^65^ Participants had a mean STAI-S score of 57.4 and a mean STAI-T score of 61.1 at baseline. In patients receiving MDMA, mean STAI-T scores decreased 1 month after the second psychotherapy session by –23.5 compared to placebo. However, these differences were not significant because of an outlier (*P*=0.0558). When the placebo outlier was removed from the analysis, STAI-T change scores between groups were statistically significant (*P*=0.0066). To examine long-term follow-up at 6 and 12 months, a crossover was done for participants in the placebo group, and the overall ANOVA for STAI-T and STAI-S showed statistically significant reductions in scores for all participants.^65^

### Psilocybin for Generalized Anxiety Disorder and Anxiety Associated With Life-Threatening Cancer

A 2011 double-blind, placebo-controlled study examined the safety and effectiveness of psilocybin (0.2 mg/kg) in 12 adults (1 male, 11 female) with a diagnosis of anxiety associated with life-threatening cancer, generalized anxiety disorder, or adjustment disorder with anxiety.^69^ Subjects received niacin (250 mg) in a separate session to act as their own controls. The STAI scores revealed no significant changes from day 1 to 2 weeks after treatment, although a nonsignificant but considerable decrease for STAI-S was seen 6 hours after administration. No significant STAI-S anxiety change was observed, but a decrease in STAI-T scores was sustained during the 6-month follow-up. A significant decrease was observed at the 1- and 3-month follow-ups after the second treatment.^69^

### Safety and Tolerability

Not every study included in this review reported data regarding adverse effects. For the studies that did, the incidence of adverse effects is summarized in Table 2. Overall, ayahuasca, ketamine, LSD, MDMA, and psilocybin were well tolerated. The most common adverse effects were transient elevated blood pressure, nausea/vomiting, dissociation/derealization, and transient anxiety/distress. Although increases in blood pressure were reported for all psychedelics other than MDMA, only ayahuasca, LSD, and 1 ketamine trial produced clinical hypertension in some participants. Ayahuasca produced nausea/vomiting, as well as transient increase in blood pressure. Ketamine had the highest rates of drowsiness and dissociation/derealization. LSD caused increased cold sensitivity and transient anxiety. MDMA had the highest rate of headaches. An adverse effect that was unique to MDMA was jaw clenching. Along with the other adverse effects listed in Table 2 for the MDMA trial, no intervention was needed and all resolved after the session.^65^ Although no other adverse effects were identified by the authors of the psilocybin trial, psilocybin produced a statistically significant elevation in blood pressure.^69^ The mean elevated systolic blood pressure of patients after taking psilocybin was 138.9 mm Hg, which is not considered to be hypertensive, compared to the mean of 117.0 mm Hg during the niacin placebo sessions.^69^ All adverse effects of the psychedelics resolved after the acute drug sessions ended. No cases of prolonged hallucinations and altered state of consciousness were reported.

**Table 2.** Summary of Safety and Tolerability: Incidence of Adverse Effects

Study	Drug	Nausea/ Vomiting, %	Vision Changes, %	Drowsiness, %	Headache, %	Transient Elevated Blood Pressure, %	Dizziness, %	Dissociation/ Derealization, %	Feeling Cold/ Sensitive to Cold, %	Transient Anxiety/ Distress, %	Hallucinations, %
Dos Santos et al, 2021^70^	Aya	23.5/17.6	–	11.8	11.8	29.4^a^	–	5.9	–	–/5.9	–
Glue et al, 2017^62^	Ket	16.7/–	–	–	–	100	–	100	–	–/16.7	–
Glue et al, 2020^64^	Ket	33.3/16.7	91.7	66.7	–	100	–	100	–	–	–
Glue et al, 2018^63^	Ket	25.0/–	22.0	–	–	100	40	50	–	–	–
Taylor et al, 2018^68^	Ket	16.7/–	33.3	–	–	11.1^a^	33.3	38.9	–	11.1/–	16.7
Gasser et al, 2014^67^	LSD	–	0	–	–	4.5^a^	–	9.1	45.4	22.7/36.4	4.5
Gasser et al, 2015^66^	LSD	–	–	–	–	–	–	–	–	–	–
Wolfson et al, 2020^65^	MDMA	23.1/–	–	–	61.5	–	–	–	15.4	23.1/–	–
Grob et al, 2011^69^	Psi	–	–	–	–	100	–	–	–	–	–

^a^Hypertension defined as blood pressure >140/90 mm Hg.

Note: Missing/unavailable data are indicated with a dash (–).

Aya, ayahuasca; Ket, ketamine; LSD, lysergic acid diethylamide; MDMA, 3,4-methylenedioxymethamphetamine; Psi, psilocybin.

### Bias Assessment

The level of bias across the studies varied greatly, the highest being in the performance, selection, and detection bias domains.^61^ Table 3 presents the results of author NR's bias assessment. A detailed explanation of the assessment of each publication is provided in the Appendix.

**Table 3.** Bias Assessment Judgment for Each Domain by Study

Bias Domain	Dos Santos et al, 2021^70^	Glue et al, 2017^62^	Glue et al, 2020^64^	Glue et al, 2018^63^	Taylor et al, 2018^68^	Gasser et al, 2014^67^	Gasser et al, 2015^66^	Wolfson et al, 2020^65^	Grob et al, 2011^69^
Random sequence generation (selection bias)	Low	High	Unclear	High	Low	Unclear	Unclear	Low	Unclear
Allocation concealment (selection bias)	Low	High	Low	High	Low	Low	Low	Low	Low
Blinding of participants and researchers (performance bias)	High	High	High	High	High	High	High	High	High
Blinding of outcome assessment (detection bias)	High	High	High	High	High	High	High	High	High
Incomplete outcome data (attrition bias)	Low	Low	Low	Low	Low	Low	Low	Low	High
Selective reporting (reporting bias)	Low	Low	Low	Low	Low	Low	Low	Low	Low
Other bias	Unclear	Unclear	Unclear	Unclear	Unclear	Unclear	Unclear	Unclear	Unclear

Selection bias was assessed according to 2 factors: sequence generation and allocation concealment. The risk of bias was low if the patients were selected through a randomized process and the details of the randomization methodology were provided. If patients were randomized but the description of the methodology was insufficient, the risk was unclear. Nonrandomized trials were high risk. Allocation concealment was present in all but 2 trials, and these studies were assessed as low risk. The 2 open-label studies were assessed as high risk.

Blinding of participants and researchers, a form of performance bias, was assessed universally as high risk because each trial was either open label or participants were able to correctly guess whether they had received active treatment or placebo regardless of blinding because of the psychoactive effects of the treatment.

Blinding of outcomes, a form of detection bias, was assessed as high risk when the rater was the participant or the clinician observing the participant, for the same reason as above.

Incomplete outcome data, a source of attrition bias, were measured in terms of completion rate and completeness of outcome data. The completion rate was high (75% to 100%) in all but 1 trial, and these studies were assessed as low risk. The lower completion rate for 1 trial (66%), was assessed as high risk; however, the completion rate was likely attributable to the terminal disease in the participants.

Selective reporting, a form of reporting bias, was not observed in any of the trials. For this domain, studies were assessed as low risk because primary and secondary endpoints as well as adverse effects were included. Other biases—ranging from comorbidities, drug-drug interactions, crossover study design, and patients asking to adjust their dose according to their underlying anxiety disorder—were widely observed. For this domain, studies were assessed as having an unclear risk of bias.

The highest contributor to bias across all studies was the lack of adequate blinding of participants and researchers, resulting in performance bias. This problem is common among double-blind controlled trials that involve psychoactive compounds. Although 1 study used a low-dose anxiolytic as a placebo, the authors recognized that using an active placebo might affect the validity of their results.^64^

Additionally, many participants had a history of comorbid psychiatric diagnoses, most commonly major depressive disorder. Glue et al hypothesized that such a medical history could represent a phenotype exceptionally responsive to psychedelic treatments.^62-64^ Other sources of bias were nonrepresentative population selection, methodology of obtaining outcome data, presence of a life-threatening or terminal illness, and variable frequency and duration of follow-up with participants.

## DISCUSSION

The principal finding of our review is that ketamine, LSD, MDMA, and psilocybin decrease anxiety scores and/or the negative effects of anxiety and are well-tolerated.^62-69^ Although all studies other than the trial of ayahuasca^70^ found decreased anxiety after treatment, not all studies had significant results, likely because of the small population sizes.

### Therapeutic Effects of Psychedelics

Eight of the studies included in this review demonstrated reduced scores on anxiety outcome measures after therapy with psychedelics. The exception was the ayahuasca study by Dos Santos et al.^70^ Although the anxiety scores of the patients treated in the Dos Santos et al trial did not decrease after ayahuasca therapy, the participants reported improved self-perception and speech performance. These results still have clinical significance, as a core part of social anxiety disorder is a negative cognitive bias in self-perception. Current first-line treatments for social anxiety disorder include exposure-based cognitive behavioral therapy and SSRIs. Because patients with social anxiety disorder often still experience symptoms with these treatments, ayahuasca could potentially increase patients’ self-confidence, enhancing other treatments such as cognitive behavioral therapy.

The pattern of anxiety score improvement with ketamine was similar to the results of prior ketamine studies of patients with treatment-resistant depression.^64,71^ These randomized controlled trials that successfully used ketamine for treatment-resistant depression led to US Food and Drug Administration approval in 2019 of the ketamine *S*-enantiomer (esketamine) for treatment-resistant depression and depression with acute suicidal ideation or behavior.^72^ The 0.25 mg/kg dose appears to be the threshold dose.^62-64^ No study of the effects of ketamine on anxiety ratings thus far has examined a dose >1 mg/kg, but in the studies we reviewed, 1 mg/kg produced the best anxiolytic effects,^62-64,68^ and ketamine produced anxiety relief within 1 hour of administration.^62-64^

LSD decreased anxiety ratings and had a positive psychological effect on subjects, including increased relaxation, equanimity, and mental strength. Psychotherapy with LSD-like hallucinations may successfully relieve anxiety symptoms by breaking up a fixed pattern of thinking that is influenced by emotional bias.^66^ Gasser et al demonstrated that LSD can increase mental flexibility during psychotherapy.^66^

MDMA has the potential for long-term benefits of at least 1 year beyond administration, as the decreased scores on the STAI-S and STAI-T anxiety rating scales were maintained at the 6- and 12-month follow-ups in the Wolfson et al study.^65^ Patients also demonstrated increased coping mechanisms, determined subjectively through their greater emotional and functional quality of life. MDMA may benefit patients experiencing anxiety who are overcoming an illness.^65^

Grob et al reported a sustained reduction in anxiety with psilocybin.^69^ Scores on the STAI-T continued to decrease for the entire 6-month follow-up, reaching significance at the 1-month and 3-month follow-ups. The study authors hypothesized that the participants had decreased stress and anxiety over time.^69^

As previously mentioned, the first line of treatment for anxiety is SSRIs or SNRIs that take 4 to 6 weeks to take effect**.**^15^ Additionally, side effects such as sexual dysfunction and increased anxiety with initial use often hinder people from continuing these medications.^73,74^ The ability for the anxiolytic effects of psychedelics to manifest as early as 1 day potentially suggests a quicker alternative to mainstream treatment, especially for treatment-resistant patients. Additionally, many of the anxiolytic effects were sustained for 2 weeks. The long-lasting effects could potentially increase compliance, as patients would not be required to take daily medication.

### Safety and Tolerability of Psychedelics

In the studies we reviewed, treatment with psychedelics was generally well tolerated. The most common adverse effects were transient elevated blood pressure, nausea/vomiting, dissociation/derealization, and transient anxiety/distress.

Two patients in the ayahuasca trial reported headaches that went away by the following day without intervention. One participant experienced increased distress but was reassured by researchers about transient effects and remained calm for the remainder of the experiment. No complaints were made about the tolerability of the experience.^70^

The 1 mg/kg dose of ketamine appeared to cause the most adverse effects. After this dose, 2 subjects from the Glue et al 2017 trial^62^ felt out of control (categorized as transient anxiety/distress on Table 2), but no intervention was needed. Two adverse events that are not noted in Table 2 occurred during the Glue et al 2018 trial but were determined to be attributable to secondary causes rather than the experimental drug.^63^ One patient experienced hypertension and delirium within 5 minutes of dosing that resolved within 15 minutes and returned to baseline by 60 minutes. This event was presumed to be attributable to inadvertent IV injection. A second patient who developed a pulmonary embolism had recently started taking an oral contraceptive.^63^ The dissociative effect, the most common effect of ketamine, appeared to disappear with time and maintenance of treatment.^62-64,68^

Although some patients (22.7%) experienced transient anxiety during the Gasser et al experimental session with LSD, more participants in the placebo group (50%) reported transient anxiety.^67^ A similar pattern was found for emotional distress; 36.4% of patients in the LSD group reported emotional distress vs 33.3% in the placebo group.^67^

In the Wolfson et al study, MDMA produced few psychiatric adverse events and no reports of suicidal ideation or behavior which are concerns associated with the drug.^65^

Psilocybin did not cause any clinically concerning cardiovascular sequelae in the Grob et al study. Minor increases in blood pressure were judged to be evidence of a mild adrenergic effect.^69^

Although these studies show promising evidence of safe anxiety relief with psychedelics, the psychedelics were administered under controlled conditions in each of these studies. The risk for adverse effects potentially increases when the dose and context of the psychedelic are not controlled.

A concern associated with the legalization and safety of psychedelics is the potential for addiction. Although research is limited, LSD and psilocybin have not been shown to lead to physiologic dependence because they produce fewer reinforcing effects compared to highly addictive drugs such as cocaine.^41,75,76^ Ayahuasca has been used for physiologic dependence recovery and has not been found to have addictive properties.^77^

Ketamine and MDMA have been determined to have more potential for addiction than LSD, psilocybin, and ayahuasca because of increased positive reinforcement compared to the other psychedelics.^78-80^ When used outside of a controlled setting, ketamine has the potential to lead to cravings and withdrawal.^81^ The evidence is conflicting regarding the potential for MDMA to lead to addiction. Studies of MDMA dependence describe withdrawal effects after use,^79,82^ but whether the reaction is withdrawal or the subacute *comedown* that people experience after using the drug is unclear.^83^ Although some of the psychedelics have a possible risk of addiction, other highly addictive drugs such as benzodiazepines and amphetamines have been successfully used for medical treatment under the guidance of a medical professional.

When taken in inappropriate doses, the psychedelics discussed in this study can cause side effects such as dehydration, confusion, hypertonia, and hyperthermia because of their serotonergic properties.^84^ An additional risk is the experience of overwhelming distress, often called a “bad trip,” with these psychedelics. Adverse effects associated with a bad trip include increased anxiety and self-harm.^85^ Researchers have attempted to determine clinical features that can predict a poor reaction to psychedelics, in particular LSD, but no conclusions have been reached.^86^

Despite the reported risks, LSD has been studied for its anti-addiction properties in the treatment of alcohol use disorder. A pooled analysis of 6 randomized controlled trials found that a single dose of LSD had a statistically significant benefit on alcohol misuse at the first follow-up assessment.^87^ Ketamine has also been studied in the treatment of alcohol use disorder. In a 2022 study, patients who received 3 ketamine infusions had more days abstinent from alcohol in the 6-month follow-up period than the patients who were randomized to placebo.^88^

### Limitations

This research has several limitations. The number of studies investigating psychedelics in patients with anxiety disorders was limited. Our literature search, including the manual search, yielded only 9 studies that met our inclusion criteria, limiting the types of anxiety disorders and psychedelics we could assess. Ketamine was the only psychedelic substance with 4 studies, while LSD, ayahuasca, psilocybin, and MDMA only had 1 or 2 studies. Additionally, the results of all these studies were self-reported, making their results prone to response bias, and they used restrictive numeric self-reporting scales. As shown in Table 3, many studies had high bias ratings. The high bias ratings may be related to the legislation regulating the use of psychedelics that therefore limits study designs. In congruence with the limited study designs, each study had a small population size (n=10 to 20), affecting significance and decreasing internal and external validity, as many of the participants had life-threatening diseases. Although some of the studies were double-blind, randomized, placebo-controlled trials, the effects of the drugs are often difficult to mimic with placebo, making it easier for participants to tell they are receiving the tested psychedelic. Some of the studies did not control for depression or mention coexisting depression. As depression commonly coexists with anxiety, some of the participants may have had undiagnosed or undisclosed comorbid depression. Patients with life-threatening illnesses are likely to have comorbid depression, as changes in the progression of the illness can impact their mental health, skewing the results.

### Direction for Future Research

Future research investigating psychedelics as a therapeutic treatment for anxiety disorders should include double-blind, randomized, placebo-controlled clinical trials with large populations. Improvements in study design and more extensive trials will become increasingly more accessible as the laws in the United States and internationally open to the use of psychedelics. Additionally, when examining the effect of psychedelics on anxiety, controlling for coexisting depression will be essential. Future research can focus on determining precise dosing and frequency of administration. Another important determination is whether the psychedelic substances have a tolerance effect or if patients need less at each administration. Additional studies could investigate the effects of psychedelics such as peyote and ibogaine on anxiety because these substances have been found to have a better safety profile than many major legal and illegal drugs^89^ and have little physical dependence.^76^

## CONCLUSION

This literature review examined psychedelic use for generalized anxiety disorder, social anxiety disorder, and anxiety associated with a life-threatening disease. Ketamine, LSD, MDMA, and psilocybin consistently produced an anxiolytic effect sustained for at least 2 weeks after the therapeutic session. Although ayahuasca did not produce a significant change in anxiety ratings, patients with social anxiety disorder had increased self-perception and performance. No life-threatening psychedelic-related adverse effects were reported in any of the trials. SSRIs and SNRIs are the current pharmacologic gold standard treatment for anxiety disorders but are associated with multiple adverse effects and poor adherence. Psychedelics may represent a possible treatment option for people who are unable to tolerate the effects of the first-line medications or are unresponsive to their effects. Because much of this research is novel and conducted in small populations and in controlled settings, large double-blind, randomized, placebo-controlled trials with long-term follow-up and an examination of dosing and frequency need to be done to determine efficacy and safety.

## Notes

*This article meets the Accreditation Council for Graduate Medical Education and the American Board of Medical Specialties Maintenance of Certification competencies for Patient Care and Medical Knowledge.*

## Appendix

**Appendix.** Support for Bias Assessment Judgment for Each Domain by Study Presented in Table 3

Bias Domain	Dos Santos et al, 2021^70^	Glue et al, 2017^62^	Glue et al, 2020^64^	Glue et al, 2018^63^	Taylor et al, 2018^68^	Gasser et al, 2014^67^	Gasser et al, 2015^66^	Wolfson et al, 2020^65^	Grob et al, 2011^69^
Random sequence generation (selection bias)	Simple randomization performed by researcher who did not participate directly in the experimental sessions or have access to the raw data	Nonrandomized	Active placebo randomly injected into ketamine vials	Nonrandomized	Randomization conducted by Yale Investigational Drug Service and kept separate from investigators	Randomization methodology not mentioned	Randomization methodology not mentioned	Randomized using web-based randomization system with unique container numbers; randomization monitored by individuals without communicating with site staff, study monitors, or analysts	Order in which subjects received the treatments randomized and known only by the research pharmacist; methodology not mentioned
Allocation concealment (selection bias)	Pharmacist-prepared solution intended to closely mimic color, smell, and taste of ayahuasca; all participants naive to ayahuasca	Open label	Psychoactive-controlled, identical vials	Open label	Placebo-controlled with normal saline	Active placebo of LSD; capsules of identical size, color, and shape and bottled in sequentially numbered containers	Active placebo of LSD; capsules of identical size, color, and shape and bottled in sequentially numbered containers	Placebo was 125 mg lactose prepared by pharmacist in identical gelatin capsules	Placebo was 250 mg niacin in identical clear capsules
Blinding of participants and researchers (performance bias)	Volunteers and researchers were right about what had been ingested in 100% of the sessions	Not blinded	After ketamine dosing, all subjects reported dissociative symptoms; minimal effects for midazolam	Not blinded	17 of 18 patients correctly identified receiving ketamine	All participants correctly guessed the LSD dose administered	All participants correctly guessed the LSD dose administered	Not reported	Drug order almost always apparent to subjects and investigators
Blinding of outcome assessment (detection bias)	Participants and researchers correctly guessed what they received	Participant rated	Participant rated	Participant rated	Participant and clinician rated	Participants and independent clinician raters correctly guessed what they received	Participants and independent clinician raters correctly guessed what they received	Participant and independent rater blinded	Participant and investigator rated; almost always apparent whether psilocybin or placebo
Incomplete outcome data (attrition bias)	100% completion rate; primary outcome analysis missing data of 2 in ayahuasca group and 1 in placebo group	100% completion rate without missing data	92% completion rate without missing data	90% completion rate without missing data	94% completion rate without missing data	75% completion rate without missing data	83% completion rate; 1 audio recording and 1 questionnaire missing	94% completion rate without missing data	66% completion rate
Selective reporting (reporting bias)	All prespecified outcomes reported	All prespecified outcomes reported	All prespecified outcomes reported	All prespecified outcomes reported	All prespecified outcomes reported	All prespecified outcomes reported	All prespecified outcomes reported	All prespecified outcomes reported	All prespecified outcomes reported
Other bias	Nonrepresentative population (mostly undergraduate female students); "unnatural" context of the speech test	Almost all patients had prior MDD; none depressed at time of testing, but MDD history could represent a clinical phenotype responsive to ketamine	Most patients had prior MDD; none depressed at time of testing, but MDD history could represent a clinical phenotype responsive to ketamine	Four patients requested twice-weekly dosing for early recurrence of anxiety (usually within 4 days of dosing), and 16 received once-weekly dosing	Not mentioned	Participants had grave somatic diseases, and the course of somatic illness (worsening or improving) can impact psychological parameters independent of the intervention and contribute to missing data	No control group for the follow-up because of the crossover design	Degree of group differences impacted by an outlier in the placebo group who responded exceptionally well to psychotherapy alone compared to other participants in the placebo group during the blinded segment; potential placebo effect because 2 of 3 placebo participants believed they were assigned MDMA during the blinded segment	Variability in the extent of contact with subjects after treatment; ad hoc communication depended on the needs and wishes of subjects, some of whom were near death while others were more functional

LSD, lysergic acid diethylamide; MDD, major depressive disorder; MDMA, 3,4-methylenedioxy-methamphetamine.
